# Self-concept clarity: a comprehensive and integrative review

**DOI:** 10.3389/fpsyg.2026.1822881

**Published:** 2026-06-26

**Authors:** Srikant Manchiraju

**Affiliations:** Jim Moran College, Florida State University, Tallahassee, FL, United States

**Keywords:** culture, identity, parenting, self-concept clarity, self-esteem, self-structure, well-being

## Abstract

Self-concept clarity (SCC) refers to the extent to which individuals’ self-beliefs are clearly and confidently defined, internally consistent, and stable over time (Campbell et al., 1996). This review synthesizes conceptual, methodological, and empirical work on SCC and situates it within the broader science of the self. Its distinctive contribution is twofold. First, I sharpen the conceptual boundaries of SCC by treating it explicitly as a structural, metacognitive property of the self-concept, a judgment about the form of self-knowledge rather than its content or evaluative tone, and I disentangle the terms self-concept, self-beliefs, self-knowledge, and identity that are often used interchangeably. Second, I organize a large and uneven literature around an integrative framework that distinguishes the roles SCC can play: as an outcome of developmental, familial, cultural, and digital antecedents; as a predictor of adjustment; as a mediator of those antecedents’ effects; and as a moderator that buffers stress. Within this framework I review the measurement of SCC and offer a critical appraisal of its near-total reliance on self-report; evaluate evidence linking SCC to well-being, self-esteem dynamics, emotion regulation, interpersonal functioning, and psychopathology, attending to the strength of evidence and to inconsistencies; examine lifespan development, family socialization, and cultural variability; and consider cognitive, motivational, social, and neurobiological mechanisms. I close by identifying conceptual and methodological limitations, including mono-method assessment, unresolved causal ordering, sparse cross-national validation, and the open question of domain-specificity, and by proposing priorities for a cumulative science of SCC.

## Self-concept clarity in the science of self and identity

Questions about how people understand who they are lie at the heart of research on the self. Since the resurgence of self research in the 1960s and 1970s, the self has functioned less as a single construct than as an umbrella term spanning a family of related phenomena. Baumeister (2010) organizes this family into three broad functions: the self as a body of reflexive self-knowledge (self-concept, self-awareness, self-esteem), the interpersonal self that is constructed and maintained through roles and relationships, and the executive or agentic self that makes choices and exercises self-regulation. A long list of cognate terms populates this domain, among them self-appraisal, self-construal, self-perception, self-presentation, and self-esteem, and a recurring task for the field has been to specify how any one of them relates to the others (Baumeister, 2010; Marsh and Martin, 2011). Self-concept clarity (SCC) is best understood against this backdrop, as one facet of how people represent and stabilize knowledge about themselves.

Identity theories inspired by Erikson (1968) and operationalized by Marcia (1966) emphasized exploration and commitment in domains such as occupation, ideology, and relationships. Work on the self-concept, in parallel, examined the content of self-beliefs (e.g., traits, roles, values) and their evaluative tone (e.g., self-esteem). More recently, attention has turned to the structure and epistemic quality of self-knowledge, that is, how clearly, coherently, and stably self-beliefs are held. SCC names precisely this structural quality. Campbell et al. (1996, p. 141) defined it as “the extent to which the contents of an individual’s self-concept are clearly and confidently defined, internally consistent, and temporally stable.” SCC thus concerns the clarity, coherence, and stability of self-beliefs rather than their specific content or positivity. Two people may endorse very different traits yet both display high SCC if their self-views are well articulated and consistent; conversely, a person may feel generally positive about the self yet remain vague or conflicted in specific self-beliefs, indicating low SCC (Campbell, 1990).

A persistent source of imprecision in this literature is terminological. Terms such as self-beliefs, self-views, self-knowledge, and self-concept are frequently used as near-synonyms, and SCC is sometimes discussed alongside identity as though the two were interchangeable. I adopt the following working distinctions throughout. The self-concept is the cognitive representation of the self, the contents of self-knowledge, including traits, roles, and values, and self-beliefs and self-views are the constituent elements of that representation (Lodi-Smith and DeMarree, 2017). SCC is not additional content; it is a second-order, metacognitive property of that representation, a judgment about how clear, coherent, and stable the contents are (Dunlop, 2017). Identity is the broader, more value-laden sense of who one is and how one fits within social and cultural contexts; SCC overlaps with the clarity or commitment facet of identity but is narrower and less domain-bound (Luyckx et al., 2008; Meeus et al., 1999). Keeping the structure-content distinction in view is essential, because much of the confusion about what SCC predicts, and why, follows from conflating a property of self-knowledge with its substance.

This review pursues three objectives. First, it clarifies the conceptualization of SCC, fixes its terminological boundaries, and distinguishes it from neighboring constructs. Second, it organizes the empirical literature using an integrative framework that specifies the distinct causal roles SCC can occupy, namely outcome, predictor, mediator, and moderator, rather than cataloguing correlations without regard to their direction. Third, throughout, it moves beyond description to weigh the strength of evidence, note inconsistencies, and identify gaps. In doing so the review aims to do more than summarize: it offers a framework within which a fragmented body of findings can be compared, and it draws attention to recent work on family socialization, the digital environment, neurobiology, and cross-cultural structure that has not yet been integrated into general accounts of SCC.

## Conceptualization and measurement of self-concept clarity

### Definition and core components

The most widely cited definition of SCC (Campbell et al., 1996, p. 141) highlights three components. Clarity and confidence refer to the perceived articulateness and certainty of self-knowledge; individuals high in SCC experience a well-articulated sense of who they are, whereas those low in SCC experience chronic self-doubt (Campbell, 1990). Internal consistency denotes the degree to which self-beliefs cohere rather than contradict one another. Temporal stability captures the extent to which self-views remain relatively constant across time and situations, providing a sense of continuity. These components are conceptually separable, a point I return to as a measurement problem below, because the dominant scale treats them as a single dimension.

### Distinguishing self-concept clarity from related constructs

SCC sits among several related constructs, and its value depends on being clearly differentiated from them (Table 1). It differs from self-esteem, the evaluative dimension of the self, that is, how much individuals like or value themselves (Rosenberg, 1965). SCC concerns the structure and certainty of self-beliefs irrespective of their valence (Campbell et al., 1996). The two are positively correlated yet distinguishable: SCC explains unique variance in adjustment beyond self-esteem (Campbell, 1990), and individuals may in principle combine high SCC with low self-esteem (clearly negative self-views) or low SCC with high self-esteem (globally positive but poorly articulated self-views).

**Table 1:** Self-concept clarity compared with related self-constructs.

Construct	Core focus	Primary aspect of the self	Relation to SCC
Self-concept clarity (SCC)	Clarity, internal consistency, and temporal stability of self-beliefs	Structure/metacognition	Focal construct; a judgment about the form of self-knowledge
Self-concept	The contents of self-knowledge (traits, roles, values)	Content	SCC is a structural property of the self-concept, not its content
Self-esteem	Global evaluation of self-worth	Evaluation/valence	Correlated yet distinct; SCC concerns structure irrespective of valence
Self-concept differentiation (SCD)	Variation in self-description across roles	Structure	Often inversely related, but predicts outcomes independently of SCC
Self-complexity	Number and differentiation of distinct self-aspects	Structure	Compatible with high or low SCC depending on integration
Identity commitment	Firmness of choices in central life domains	Content/process	Overlaps with SCC but is narrower and more domain-specific
Self-concept consistency	Cross-role and cross-time similarity of self-views	Structure	A behavioral analogue of the consistency component of SCC
Self-knowledge accuracy	Correspondence of self-views with objective/other indicators	Accuracy	Independent of SCC; self-views can be clear yet inaccurate

SCC, self-concept clarity; SCD, self-concept differentiation. The primary-aspect column indicates whether each construct concerns the structure, content, evaluation, or accuracy of self-knowledge.

SCC is also distinct from self-concept differentiation (SCD), the extent to which individuals describe themselves differently across roles (Donahue et al., 1993). Although high SCD often accompanies lower SCC, the two are not interchangeable: people may hold somewhat different role-based selves yet experience them as integrated, maintaining high SCC (Sheldon et al., 1997), and SCC predicts well-being independently of SCD (Bigler et al., 2001). SCC overlaps with identity commitment, the sense of having made stable choices in central life domains (Meeus et al., 1999), but is broader and less domain-specific than the exploration and commitment processes emphasized in identity-status models (Luyckx et al., 2008). It is related to self-complexity, the number and differentiation of distinct self-aspects (Linville, 1985, 1987), but high self-complexity can co-occur with either high or low SCC depending on whether the aspects are integrated or fragmented. Finally, SCC does not require self-knowledge accuracy: individuals may hold clear but inaccurate self-views (Vazire and Mehl, 2008). Across these comparisons, the organizing principle is the same: SCC is a meta-structural judgment about the form of self-knowledge, not a statement about its content, accuracy, or positivity.

### Dimensionality and domain specificity

Two structural questions about SCC remain only partly resolved. The first is dimensionality. Although the construct is defined by three components, the Self-Concept Clarity Scale (SCCS) is typically treated as unidimensional, and factor-analytic work generally supports a single dominant factor (Campbell et al., 1996; Cicero, 2020). Yet the definitional separability of clarity, consistency, and stability sits uneasily with a strictly unidimensional measurement model, and few studies have tested whether the components carry distinct predictive weight. This is less a settled fact than an underexamined assumption. The second question is domain specificity. By analogy to the self-concept itself, which is reliably multidimensional (the five-factor AF5 model distinguishes academic, social, emotional, family, and physical self-concept and replicates across the United States, Brazil, and China; Chen et al., 2020; García et al., 2013, 2018), one might ask whether clarity is similarly domain-specific. Domain-specific clarity scales for vocational and other identity domains suggest that it can be (Luyckx et al., 2008; Vignoles et al., 2006), yet the SCCS is almost always administered as a global index. Whether a person can be clear about who they are as a professional while remaining unclear as a partner, and which domain-specific clarities matter for which outcomes, is largely unaddressed and is a substantive gap rather than a technicality.

### The self-concept clarity scale and its validity

The dominant measurement tool is the SCCS (Campbell et al., 1996), a 12-item self-report instrument rated on Likert-type scales. Items tap perceived clarity, consistency, and stability (e.g., “In general, I have a clear sense of who I am and what I am”; reverse-scored “My beliefs about myself often conflict with one another”). Internal consistency typically ranges from about 0.80 to 0.90, the factor structure is primarily unidimensional, and test–retest correlations over weeks to months are substantial (Campbell et al., 1996; Cicero, 2020). Validity evidence is, on its face, robust. SCC correlates positively with self-esteem, identity commitment, and self-concept stability, and negatively with neuroticism, trait anxiety, and rumination (Campbell, 1990; Smith et al., 1996). It is discriminable from self-esteem and SCD, predicting unique variance in depression, life satisfaction, and coping (Bigler et al., 2001; Campbell et al., 1996). Prospectively, lower SCC forecasts later internalizing symptoms (Van Dijk et al., 2014), and changes in identity clarity track changes in depressive symptoms across adolescence and the twenties (Luyckx et al., 2013).

### A critical appraisal of self-concept clarity measurement

These strengths notwithstanding, the evidentiary base for SCC rests on a narrow methodological base that warrants more scrutiny than it usually receives. First, assessment is almost entirely mono-method: SCC is measured by self-report, and so are most of its correlates. Shared method variance is therefore a live alternative explanation for a non-trivial portion of the SCC-adjustment associations, and the problem is compounded when both SCC and outcomes such as depression are introspective judgments colored by current mood. Second, self-report presumes metacognitive access that low-SCC individuals may, by definition, lack; a person confused about who they are may not accurately report how confused they are. Multi-method assessment, including informant reports, reaction-time and experience-sampling indices of self-belief accessibility and day-to-day variability, and narrative coding of autobiographical coherence, exists but is used too rarely to triangulate the construct (Diehl et al., 2001; McLean et al., 2007; Vazire and Mehl, 2008). Third, comparative evaluation of alternative measures is thin: behavioral, implicit, and narrative analogues are typically reported in isolation rather than benchmarked against the SCCS, so their convergent and incremental validity is unclear. Fourth, the cross-cultural psychometrics of the SCCS are underdeveloped. Measurement invariance has been established across race and sex within largely U.S. samples (Cicero, 2020), but rigorous cross-national invariance testing is sparse, which matters because cross-cultural claims about SCC presuppose that the scale measures the same thing everywhere. Until these gaps are addressed, confident causal and cross-cultural conclusions outrun the measurement foundation on which they rest.

### An integrative framework for organizing self-concept clarity research

**Figure 1:**
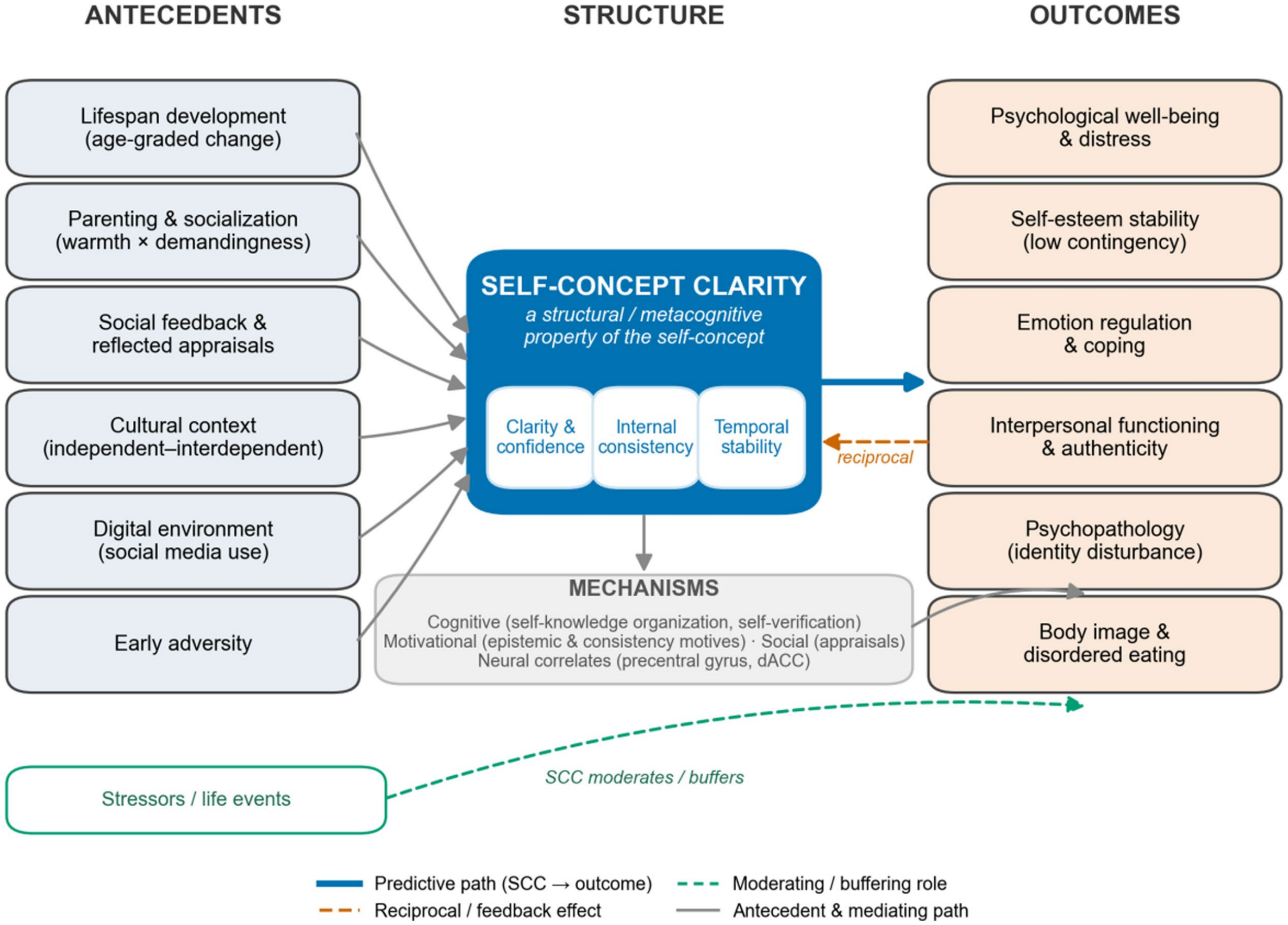
An integrative framework for self-concept clarity: antecedents, structure, mechanisms, and outcomes. Self-concept clarity (SCC) is modeled as a structural property of the self-concept that develops out of antecedents (left), predicts adjustment outcomes (right) directly and through cognitive, motivational, social, and neural mechanisms, exhibits reciprocal feedback with outcomes, and buffers the impact of stressors.

A recurring difficulty in summarizing the SCC literature is that the same association is interpreted, across studies, as evidence that SCC causes an outcome, that the outcome shapes SCC, or merely that the two co-occur. Much of the literature is cross-sectional, and reviews have tended to report correlations without committing to a direction. To impose order, I organize the evidence around the distinct roles SCC can occupy in a causal system (Figure 1). As an outcome, SCC develops out of antecedents: age-graded maturation, family socialization, social feedback, cultural context, the digital environment, and adversity. As a predictor, SCC forecasts adjustment outcomes such as well-being, self-esteem stability, emotion regulation, interpersonal functioning, and psychopathology. As a mediator, SCC transmits the effects of distal antecedents to those outcomes; for example, the identity-disruption account holds that early adversity erodes SCC, which in turn heightens vulnerability to appearance pressures and disordered eating (Vartanian et al., 2018). As a moderator, SCC buffers the impact of stressors on well-being, such that the self-esteem and mood of high-SCC individuals erode less following failure or conflict (Paradise and Kernis, 2002).

Two features of the framework deserve emphasis. First, these roles are not mutually exclusive, and the relations are frequently reciprocal rather than one-way. The reciprocal-effects logic established for academic self-concept and achievement, in which each causes the other over time (Marsh and Martin, 2011), almost certainly applies to SCC and adjustment: low SCC predicts later depressive symptoms, and depressed mood degrades the clarity of self-views, generating feedback loops (Van Dijk et al., 2014). Second, the framework is a diagnostic tool: it makes explicit that a given study typically licenses only one of these interpretations, and that the field’s confidence in any causal claim should track the design that produced it. I use this scheme to structure the sections that follow, treating antecedents, predictive relations, and mechanisms in turn, and flagging where the evidence is correlational, longitudinal, or experimental.

## Antecedents of self-concept clarity

### Lifespan development

SCC changes systematically across the lifespan, reflecting cognitive maturation, socialization, and role transitions. In childhood, self-concepts are concrete and behaviorally specific, and children may lack the metacognitive capacity that SCC measures presuppose (Harter, 1988, 1999). Across early adolescence, SCC often declines as youth experiment with roles and possible selves, then rises through late adolescence and emerging adulthood as identities consolidate (Arnett, 2000; Klimstra et al., 2009; Meeus et al., 1999). A meta-analytic synthesis of lifespan trajectories indicates that SCC follows a curvilinear course, rising into and peaking in midlife and somewhat lower in young adulthood and older age (Lodi-Smith and Crocetti, 2017). In later life, transitions such as retirement and bereavement can challenge SCC, though a strong narrative identity may preserve continuity (Diehl et al., 2001; McAdams, 2001). Importantly, short-term fluctuation in SCC is normative and not inherently maladaptive (Klimstra et al., 2009); it is chronic or pronounced low clarity, not momentary variability, that is associated with internalizing and externalizing problems (Van Dijk et al., 2014).

### Parenting and family socialization

Among the most theoretically tractable antecedents of SCC is the family, yet the SCC literature has typically invoked only the warmth dimension of parenting, leaving the relationship undertheorized. Socialization research has long modeled parenting along two largely orthogonal dimensions, warmth or responsiveness and demandingness or strictness, whose crossing yields four prototypical styles: authoritative (high warmth, high demandingness), authoritarian (low warmth, high demandingness), indulgent or permissive (high warmth, low demandingness), and neglectful or uninvolved (low on both; Maccoby and Martin, 1983). This two-dimensional typology provides the missing framework for relating parenting to SCC. Warm, autonomy-supportive parenting plausibly fosters the secure exploration through which a coherent self consolidates, whereas psychologically controlling, inconsistent, or rejecting parenting may generate confusion about one’s preferences and worth (Soenens and Vansteenkiste, 2010). What remains genuinely open is how the two dimensions combine: whether, for instance, the clarity costs of low warmth are amplified or offset by high demandingness. A notable gap is that the four-fold typology has rarely been examined with the SCCS directly, so claims about which parenting configurations produce clear selves are currently more inferential than evidential.

Direct longitudinal evidence on the family does exist and is instructive about causal direction. In a six-wave, multi-informant study of nearly 500 families, Crocetti et al. (2016) found that SCC was transmitted from parents to adolescents and not the reverse, an intergenerational, unidirectional pathway, with SCC increasing and becoming more rank-order stable over time. Complementary work underscores that the family domain can be a source of harm as well as support: parent–child communication and family functioning are more strongly tied to adolescents’ mental-health deterioration than broader social factors (Ertema et al., 2025). Together these findings position family socialization as a genuine developmental input to SCC rather than a mere correlate, while also indicating that its effects are not uniformly benign.

### Social feedback and reflected appraisals

Beyond the family, SCC is shaped by the consistency of social feedback. Validating, coherent appraisals from significant others support a clear and stable self-concept, whereas inconsistent, rejecting, or invalidating feedback undermines it (Harter, 1999; Swann, 1987). Stable roles and strong group identifications supply ready-made self-definitions that can be internalized (McAdams, 2001; Vignoles et al., 2006), while conflicting or stigmatized roles complicate clarity. Because reflected appraisals operate over development, they function simultaneously as antecedents of SCC and as proximal mechanisms, a duality I take up in the section on mechanisms.

### The digital environment and social media

A development largely absent from earlier reviews is the rise of social media as a context for identity. The most direct synthesis to date, a systematic review and meta-analysis by Petre (2021), found a significant negative association between Internet use and SCC (pooled r = −0.35, 95% CI [−0.44, −0.24]; 11 effect sizes, N = 3,298), with the strongest links for compulsive rather than merely frequent use and considerable heterogeneity across studies. Recent longitudinal work helps adjudicate direction and is, tellingly, more consistent with SCC as antecedent than as consequence. Using a design that separates within- from between-person effects, Zhang et al. (2025) found that lower SCC predicted later short-video addiction rather than the reverse, and Dodan and Negru-Subtirica (2025) reported that lower SCC forecast greater TikTok-related digital stress, including fear of missing out and online vigilance. SCC also appears protective: adolescents higher in SCC were less susceptible to peer pressure toward addictive social-media use (Xu et al., 2023). On balance, the evidence points to low clarity as a vulnerability for problematic use rather than to screen time as a cause of low clarity. This vulnerability reading is tentative, however, because the meta-analytic base is weighted toward cross-sectional studies and shows high heterogeneity.

### Early adversity and identity disruption

Adverse early experience is a further distal antecedent. The identity-disruption model proposes that early adversity destabilizes the self-concept, and that this disruption, indexed in part by low SCC, increases sensitivity to sociocultural appearance pressures, thereby raising risk for body dissatisfaction and disordered eating (Vartanian et al., 2018). This account is valuable because it positions SCC as a mediator linking distal adversity to a specific clinical outcome, illustrating the integrative framework in action. It also extends SCC into body image, a domain not traditionally associated with it, and invites tests of whether domain-specific clarity of the physical self is the more proximal predictor.

## Self-concept clarity as a predictor of adjustment

### Psychological well-being and distress

The most replicated finding in this literature is that higher SCC accompanies better psychological well-being: more positive affect, higher life satisfaction, and greater overall adjustment, with associations that survive controls for self-esteem and broad traits (Campbell et al., 1996; Setterlund and Niedenthal, 1993). Conversely, low SCC is associated with negative affect, depression, anxiety, and general distress (Campbell, 1990; Smith et al., 1996), and predicts increases in depressive and internalizing symptoms over time, particularly during adolescence and emerging adulthood (Luyckx et al., 2013; Van Dijk et al., 2014). The strength of this evidence is uneven, however. The concurrent associations are large and consistent but vulnerable to shared-method and mood-congruence artifacts; the prospective evidence is more compelling but concentrated in adolescent and young-adult samples and in a relatively small set of longitudinal cohorts. Claims that SCC confers well-being are therefore better supported in those developmental windows than as a general adult truth.

### Self-esteem dynamics and personality

SCC and self-esteem are intertwined at the level of both magnitude and dynamics. Beyond their moderate positive correlation, low SCC predicts more unstable, contingent self-esteem that fluctuates with situational success and failure, whereas high SCC accompanies stable, less contingent self-esteem and lower defensiveness under ego threat (Kernis, 2003; Paradise and Kernis, 2002). The theoretical account of why SCC and self-esteem are linked nonetheless remains underdeveloped, and their causal ordering is genuinely unsettled: stable self-knowledge may scaffold positive self-evaluation, positive self-evaluation may lend a sense of certainty, or a third factor such as emotional stability may drive both. By analogy to the reciprocal-effects model (Marsh and Martin, 2011), the most defensible interim position is bidirectional influence, but the field lacks the cross-lagged tests needed to confirm it (Lodi-Smith and DeMarree, 2017). At the trait level, higher SCC accompanies lower neuroticism and higher conscientiousness and extraversion (Diehl et al., 2001), and is associated with autonomous, authentic functioning (Sheldon et al., 1997). Its relation to narcissism is heterogeneous: grandiose narcissism pairs with high reported clarity, whereas vulnerable narcissism pairs with low clarity and lability (Campbell et al., 2004; Kernis, 2003), suggesting that reported SCC may differentiate adaptive from fragile self-regard.

### Emotion regulation, coping, and meaning-making

Clearer self-concepts are associated with more adaptive emotion regulation, including greater use of problem-focused coping and reappraisal and less avoidance and rumination (Diehl et al., 2001; Smith et al., 1996), and with more successful meaning-making, as narrative coherence both reflects and sustains a clear sense of self (McLean et al., 2007; Park, 2010). A well-defined self may guide regulation toward personally meaningful goals, consistent with self-regulatory accounts in which clear standards enable effective self-direction (Carver and Scheier, 1998). The directionality caveat recurs here with force: rumination degrades self-clarity even as low clarity invites rumination, and most supporting studies cannot separate the two.

### Interpersonal functioning and psychopathology

SCC has interpersonal consequences. Higher SCC accompanies more secure attachment and greater comfort with intimacy (Mikulincer and Shaver, 2005), and supports authenticity and self-disclosure (Kernis and Goldman, 2006; Sheldon et al., 1997). Lower SCC accompanies interpersonal conflict and, in romantic relationships, lower satisfaction and greater perceived unpredictability (Lewandowski et al., 2010). With respect to psychopathology, low SCC is implicated in conditions involving identity disturbance. Beyond depression and anxiety, it is conceptually and empirically central to borderline personality disorder, whose criteria include unstable self-image and chronic emptiness (American Psychiatric Association, 2013), and identity diffusion akin to low SCC appears across Cluster B pathology (Schwartz, 2001). The evidence here is strongest as a robust marker of vulnerability and weakest as a demonstrated cause; much of it is cross-sectional and clinical-correlational, and the field would benefit from prospective designs that test whether low SCC precedes, rather than merely accompanies, identity-related disorder.

## Mechanisms linking self-concept clarity to adjustment

### Cognitive mechanisms

Cognitively, SCC reflects the organization of self-knowledge in memory. High SCC corresponds to an integrated, hierarchically structured network of self-schemas that supports rapid, consistent retrieval, whereas low SCC corresponds to a fragmented network with weak or conflicting links and greater context dependency (Campbell et al., 1996). Self-verification processes reinforce these structures: individuals high in SCC seek and favor self-confirming feedback, stabilizing self-views over time, while those low in SCC are more swayed by salient recent feedback (Swann, 1987). Metacognitive certainty, that is, beliefs about the reliability of one’s self-beliefs, together with temporal integration of past, present, and future selves into a coherent narrative, further supports clarity (McAdams, 2001).

### Motivational and affective mechanisms

SCC engages epistemic motives for closure, predictability, and order (Kruglanski, 1989): clear, stable self-definitions satisfy these motives within the self-domain, whereas chronic ambiguity about identity is unsettling. Self-consistency motives press individuals to reconcile discrepant self-relevant information (Festinger, 1957), and when these efforts are overwhelmed by highly discrepant roles, clarity declines. Affect and SCC are mutually constitutive: negative mood can transiently lower clarity by foregrounding inconsistencies, and chronic low clarity heightens vulnerability to depression and anxiety, closing a reciprocal loop (Van Dijk et al., 2014).

### Social mechanisms

Socially, reflected appraisals and validating feedback from parents, partners, and friends supply and stabilize self-relevant information (Mikulincer and Shaver, 2005; Swann, 1987), and authentic, coherent self-presentation across contexts maintains clarity, whereas chronic false-self behavior erodes it (Kernis and Goldman, 2006; Sheldon et al., 1997).

### Neurobiological correlates

A small but growing neuroimaging literature has begun to identify neural correlates of SCC, though it must be read with care. Most brain research on the self concerns self-referential processing in cortical midline structures, notably the medial prefrontal cortex and the default-mode network (Gusnard et al., 2001), and bears on the self-concept in general rather than on clarity as Campbell et al. (1996) defined it. A few studies, however, measure the SCCS directly. In a large resting-state sample, higher SCC was associated with altered spontaneous activity in the right precentral gyrus and its connectivity with the inferior parietal lobe, and these features related to well-being through SCC (Xiang et al., 2022); separately, spontaneous activity in the dorsal anterior cingulate cortex moderated the association between self-esteem and SCC (Chen et al., 2022). This work is promising but preliminary, is based on a small number of largely young-adult samples, and should not be overinterpreted: the broad self-referential literature is relevant to SCC only by extension, and direct neurobiological evidence on clarity remains scarce.

## Cultural perspectives on self-concept clarity

Most SCC research has been conducted in Western, individualistic settings, raising questions of generalizability that are best addressed within an explicit cultural framework rather than as scattered caveats. Two frameworks are pertinent. Triandis (1989) distinguished private, public, and collective aspects of the self and argued that culture shapes which is chronically sampled: the private self in individualist contexts, the collective self in collectivist ones. Markus and Kitayama (1991) contrasted independent self-construals, organized around stable internal attributes expressed across situations, with interdependent self-construals, organized around relationships and responsive to context. Because the SCCS rewards perceived cross-situational consistency, it arguably encodes an independent, Western ideal of selfhood (Heine et al., 1999), and Campbell et al. (1996) indeed observed lower SCC and weaker links between SCC and self-esteem among Japanese than Canadian participants.

The inference that consistency simply does not matter outside the West is, however, too strong. In an eight-culture study, self-concept consistency predicted hedonic and eudaimonic well-being in both individualistic and collectivistic cultures, with cultural variation appearing in degree rather than direction (Church et al., 2014). A defensible reading is that some coherence of self-knowledge supports well-being broadly, even as the culturally valued form of that coherence, whether trait consistency or contextually appropriate responsiveness, differs. Consistent with this, a relational-interdependent self-construal is itself associated with greater self-concept consistency and well-being (Cross et al., 2003). Two further points sharpen the cultural analysis. First, the structure of the self-concept appears substantially invariant across cultures: the five-factor AF5 model replicates across the United States, Brazil, and China (Chen et al., 2020; García et al., 2013, 2018), which suggests that domain organization may be more culturally general than clarity norms. Second, biculturalism is a revealing case: individuals who integrate multiple cultural identities into a coherent whole report higher SCC and better outcomes, whereas fragmented or conflicted bicultural identities accompany lower SCC (Benet-Martínez and Haritatos, 2005). The cross-cultural agenda for SCC thus concerns which forms of clarity are adaptive in which settings, more than whether clarity matters at all. The field cannot answer it until the SCCS is shown to be measurement-invariant across the cultures being compared (Cicero, 2020).

## Interventions, change, and optimal self-concept clarity

Although SCC is trait-like, it is malleable. Psychotherapy and counseling often target it implicitly by helping clients articulate values and integrate conflicting self-aspects (McAdams, 2001), and identity-focused and values-clarification interventions in adolescence and emerging adulthood promote clarity by encouraging deliberate commitment (Luyckx et al., 2008; Sheldon and Elliot, 1999). Expressive and narrative writing, which integrates disparate experiences into a coherent story, plausibly strengthens SCC and yields health benefits (Pennebaker and Seagal, 1999). Mindfulness and acceptance-based approaches present a more ambiguous case: by loosening over-identification with transient states they may foster a stable yet flexible sense of self (Kabat-Zinn, 1990), yet their emphasis on a fluid, decentered self could reduce rigid clarity, and their net effect on SCC as Western psychology operationalizes it is an open empirical question. A candid appraisal is that intervention evidence specific to SCC is mostly indirect, because SCC is seldom the primary target or outcome, so claims that these approaches raise clarity are at present reasonable inferences rather than established effects.

Finally, more clarity is not unconditionally better. Rigidly held self-concepts can constrain growth and adaptability and may underwrite interpersonal difficulty or resistance to legitimate role demands (Kernis and Goldman, 2006), and clarity attached to inaccurate or maladaptive content, such as rigidly negative self-views in depression or grandiose ones in narcissism, need not be adaptive (Campbell et al., 2004; Kernis, 2003). Optimal SCC likely involves a balance between coherence and openness to revision: knowing one’s core commitments while remaining able to integrate new information.

## Limitations and directions for future research

Several limitations of the SCC literature double as priorities for future work. Conceptually, the SCCS may embed a Western bias toward consistency and stability as markers of a good self (Heine et al., 1999; Markus and Kitayama, 1991); culturally grounded conceptualizations, and demonstrations of cross-national measurement invariance, are needed before comparative claims can be trusted (Cicero, 2020). Methodologically, the near-total reliance on self-report invites multi-method triangulation through informant reports, behavioral and experience-sampling indices, and narrative coding (Diehl et al., 2001; Vazire and Mehl, 2008). The three definitional components, clarity, consistency, and stability, should be measured separately so that their distinct predictive roles, and the question of domain-specific versus global clarity, can be tested (Luyckx et al., 2008). Causal ordering remains the central unresolved issue: cross-lagged and experimental designs are required to distinguish SCC as cause, consequence, and reciprocal partner of adjustment, and the few directionally informative studies point toward reciprocal and vulnerability dynamics rather than simple one-way effects (Crocetti et al., 2016; de Moor et al., 2023; Marsh and Martin, 2011; Zhang et al., 2025). Substantively, the newest frontiers, including family-style configurations tested with the SCCS, the digital environment, neurobiology, and body image, are promising but thinly populated, and integrating SCC with narrative, social-identity, and embodied theories of the self would help move the field beyond a list of correlates toward a cumulative science.

## Conclusion

Self-concept clarity offers a valuable lens on how people organize and experience their identities. Understood as a structural, metacognitive property of self-knowledge rather than its content or valence, SCC is distinct from self-esteem, self-concept differentiation, and identity status, and it is systematically related to well-being, self-esteem stability, emotion regulation, interpersonal functioning, and psychopathology. The contribution of this review is to fix those conceptual boundaries and to organize an uneven literature around the distinct roles SCC plays, namely outcome, predictor, mediator, and moderator, so that the strength of each claim can be judged against the design that supports it. Read this way, the evidence is strongest for low SCC as a marker of vulnerability to internalizing symptoms and identity disturbance in adolescence and emerging adulthood, and weakest where causal language has outrun cross-sectional, mono-method data. The most valuable next steps are methodological and integrative: multi-method, cross-culturally invariant measurement; cross-lagged and experimental tests of direction; attention to domain-specific clarity; and the incorporation of family, digital, neurobiological, and cross-cultural evidence into a unified account. Pursued together, these steps can make SCC a bridge construct linking personality, social, developmental, and cultural perspectives within the science of self and identity.
